# The frequency of referent objects influences expectations about label length

**DOI:** 10.1016/j.actpsy.2019.04.010

**Published:** 2019-05

**Authors:** Tibor Tauzin

**Affiliations:** Cognitive Science Department, Central European University, Október 6. u. 7, H-1051 Budapest, Hungary

**Keywords:** Pragmatics, Referent frequency, Word length, Situational context, Communicative efficiency

## Abstract

Earlier studies suggest that word length is influenced by the linguistic context to be precise and concise at the same time. The present study investigates whether the referential-situational context can also have an effect on the expected length of words. To test this assumption a salient property of the situational context, that is, the frequency of the unfamiliar referents was varied. The participants watched pictures of novel objects in the observational phase, presented either frequently or rarely. In the test phase they saw the same pictures of objects one by one and were asked to select one of two unfamiliar labels, which – according to them – could be the name of the object displayed. The two labels provided for each object at test had either short or long orthographic length. It was hypothesized that participants will select the long label more frequently when they had to guess the name of rare objects in contrast to frequent ones. The findings supported this hypothesis. Rare objects were paired with long labels significantly more often than frequent objects, resulting in a significant difference also when contrasted to chance-level. The results were similar if abbreviated or completely different label pairs were presented to the participants in the test phase suggesting that the situational context is taken into account when language users infer word form.

## Highlights

• Rare referent objects are paired with longer labels in contrast to frequent referent objects.
• Referent frequency affects expected label length of novel words, at least when the referent object is unfamiliar.
• These results suggest that the referential-situational context is taken into account when word length is inferred.

## 1 Introduction

Words are considered to be purely symbolic information encoding devices that have an arbitrary relation with their referents (de Saussure, 1916/1983), as they are determined by social conventions (Peirce, 1932) in contrast to natural iconic or indexical signals. An obvious example showing the arbitrariness of words is the fact that a given referent has distinct labels in most languages. Even indexical (e.g. “this”) and iconic (e.g. “tick-tock”) words show such a difference, suggesting that arbitrary social conventions are highly relevant in word formation. Accordingly, it is a widespread assumption that the referent of a given word cannot be inferred from word form, as the relation between the two is exclusively based on acquired and intergenerationally transmitted social knowledge.

The ‘strong’ arbitrariness account, however, was challenged by the phonesthemic approach, which argues that based on form-meaning pairings language users have expectations about the referents of unfamiliar labels. For instance, in his seminal work, Köhler (1929, and later Werner, 1957, Werner & Wapner, 1952) reported that participants responded congruently when they had to pair an unfamiliar label (*takete* or *baluba*) to one of two unfamiliar referent objects that were either rounded or jagged. It was found that adults expected *takete* to refer to the jagged, while *baluba* to refer to the rounded shape. These findings were replicated in recent studies with adults (e.g. Ramachandran & Hubbard, 2001), and crucially also with 2.5-year-old toddlers (Maurer, Pathman, & Mondloch, 2006) suggesting that phonesthemes can foster language acquisition (Imai, Kita, Nagumo, & Okada, 2008; Monaghan, Mattock, & Walker, 2012) by helping to identify the referent of unfamiliar words.

More systematic linguistic studies (Bergen, 2004; Kelly, 1992) and connectionist models (e.g. Gonnerman, Seidenberg, & Andersen, 2007) revealed that the phonesthemic effect is a general principle of language. The correspondence between form and meaning can affect, for instance, the expectations about gender of unfamiliar proper names (Cassidy, Kelly, & Sharoni, 1999) or whether an unfamiliar word refers to a bird or a fish species (Berlin, 1994). These results together call into question the main assumption of the arbitrariness account and imply that word form and some characteristics of the referents can be related.

Another line of research suggests that the linguistic context, that is the units of language surrounding a given linguistic component, has a crucial effect on word form as well. Recently, Piantadosi and colleagues (Piantadosi, Tily, & Gibson, 2011) revealed that – in contrast to previous explanations based on Zipf's second law (Zipf, 1935, Zipf, 1949) – inter-word statistical dependency predicts word length in different syntactic categories. According to their result, those words are the shortest that are the most predictable based on the linguistic context, which was interpreted as a consequence of communicative efficiency to convey information in a concise but precise manner. Apart from the effect on word length distribution in the mental lexicon, the principle of communicative efficiency actively influences language production as well. Accordingly, the abbreviated (as opposed to non-abbreviated) word forms are selected by language users depending on the linguistic context (Mahowald, Fedorenko, Piantadosi, & Gibson, 2013). For example, if a sentence had been predictive of a missing word – in contrast to neutral linguistic contexts – adult participants tended to choose shorter words more frequently (among word pairs like chimp/chimpanzee or math/mathematics). These findings together were also interpreted as suggesting that signal form is not simply the result of arbitrary social conventions, but it is also influenced by contextual effects.

However, there is a disagreement between the theoretical approaches that try to explain the non-arbitrary relation between words and referents. The synesthetic view (Ramachandran & Hubbard, 2001) proposes that the relations between visual features of objects, sound contour and vocalization are determined by neural structures. This approach argues that interconnections between brain regions that process different types of information can cause synesthesia, which might serve as a basis for the evolution of proto-languages. Consequently, it was proposed that languages consist of, and are indeed based on, more iconic, and as such, causally determined elements than it was previously assumed (Perniss, Thompson, & Vigliocco, 2010). However, although neural interconnections necessarily play a role in the non-arbitrariness of word forms functional brain network topology of humans may not be sufficient to account for word-referent relations in itself as the non-arbitrary relation between wordform and meaning includes expectations about label length as well.

The convergence theory of codes (Harm & Seidenberg, 2004; Seidenberg & Gonnerman, 2000) posits that non-arbitrariness is a consequence of statistical regularities between semantics and phonology (and also: ortography). For instance, *glisten*, *glimmer* and *gleam* show similarity in both form and meaning. Therefore, it can be assumed that the relation between word form and meaning is not just causal-neuronal, but correlation-based and acquired through statistical learning. Accordingly, the convergence theory holds that the lack of arbitrariness of word form is a result of monitoring correspondence between different aspects of incoming information similarly to other knowledge domains. Statistical learning, however, does not shed light on the function of relation between form and meaning and it does not explain why and how non-arbitrariness emerges, but simply describes the existing non-arbitrary regularities between words and their meaning in the mental lexicon.

In contrast, the principle of communicative efficiency – that is to be precise and concise at the same time – may explain at least part of the correlation found between word forms and meaning as it can also affect word length among other factors. In line with the central claim of pragmatic theories of communication (Grice, 1957; Sperber and Wilson, 1986, Sperber and Wilson, 2002) an increasing number of empirical studies (e.g. Bennett & Goodman, 2018; Kanwal, Smith, Culbertson, & Kirby, 2017; Mahowald et al., 2013; Piantadosi et al., 2011) suggest that the relation between word length and meaning can be a result of communicative efficiency in a given linguistic context. Consequently, a shorter word is sufficient to produce when prior components of a sentence can help to predict the expected ending of an utterance. However, the situational context, that is the non-linguistic set of circumstances of a communicative act (e.g. physical surroundings, intentions of communicators, properties of the referents), was often neglected when the efficiency of signals was empirically investigated, despite that situational variables can be highly relevant when interpreting or producing an utterance (Tanenhaus, Spivey-Knowlton, Eberhard, & Sedivy, 1995). In fact, comprehension of word meaning relies on the appropriate identification of the referent, which can be achieved by tracking the relevant changes and features of the situational context of communication (Tauzin & Gergely, 2018).

Therefore, it can be hypothesized that at least some relevant characteristics of the situational context may also cause differences in word length. For instance, signals that represent frequent, often mentioned objects became shorter to make communication more efficient, while those that stand for rare and hardly mentioned referents remained or became longer. Thus, the principle of communicative efficiency could also affect etymology, and similarly to the linguistic context (Kanwal et al., 2017; Mahowald et al., 2013; Piantadosi et al., 2011) the relevant characteristics of the referential-situational condition may also influence word length (on a different time scale). As a consequence, a statistical relation between word length and meaning could emerge, which can be represented in the mental lexicon leading to an implicit expectation that a longer label will likely refer to a less frequent object.

To test this question in an experimental setup a set of objects were presented to the participants in a random sequence. Half of the objects were shown ten times more frequently than the other half of the objects in the same series. After such an initial exposure, participants were asked to choose a label for each object out of a short and long non-word. To avoid major biases, which can be caused by prior knowledge the depicted objects were completely novel and the non-words were unfamiliar. It was hypothesized that participants will select the long label out of the two alternative non-words more frequently when they had to guess the name of a less frequent in contrast to a more frequent referent. To measure whether this effect is a result of a tendency to abbreviate a frequent object's name (see also Mahowald et al., 2013) or can be found even in completely different alternative labels two conditions were designed with an Abbreviated Label and with Different Labels.

## 2 Methods

### 2.1 Participants

One-hundred and eighty university students participated in the experiment in exchange for a small monetary compensation (*N* = 180). All of them were native Hungarian speakers, 96 were female. They were aged between 18 and 31 years (*M* = 21.96 years, *SD* = 2.19). Participants were randomly assigned to one of the two conditions, which were equal in terms of sample size (Different Labels: *N* = 90, 46 females; Abbreviated Label: *N* = 90, 50 females). Sample size was defined a priori based on a previous study (Mahowald et al., 2013). Four additional participants were excluded, due to technical error (*N* = 3) or because the participant did not act in line with the instructions (*N* = 1). The United Ethical Review Committee for Research in Psychology of Hungary approved of the recruitment and the experimental procedure.

### 2.2 Apparatus

The experiment was run on a 15-inch MacBook Pro by PsyScope X (http://psy.ck.sissa.it). Response data were collected by a PsyScope Button Box.

### 2.3 Stimuli

During the first, observational phase the participants watched still pictures of 20 unfamiliar, greyscale objects, which were shown in a random order. Objects were created using a 3D animation software. Half of the pictures were presented 30 times (frequent objects), while the other half of the pictures were presented 3 times (rare objects). Object pictures were on screen for 1000 ms. Before the presentation of an object a fixation cross was shown for 200 ms.

**Fig. 1:**
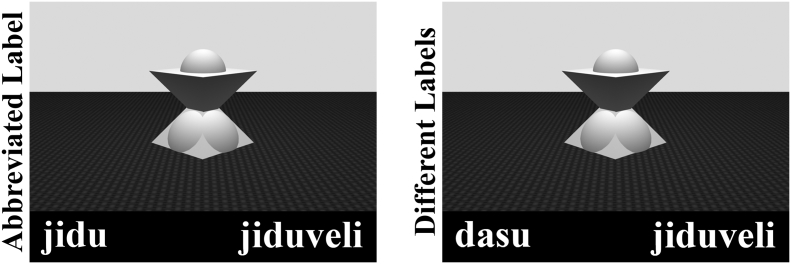
Example of a test trial in the Abbreviated Label and Different Labels conditions.

In the test phase each object was shown once to the participants one by one. Two unfamiliar non-words were displayed on the left and right side of the screen below the object's picture with short and long orthographic length (which were used to differentiate between label lengths in previous studies, see e.g. Piantadosi et al., 2011). Short non-words consisted of 4 letters, while the long non-words consisted of 8 letters. All the non-words were meaningless and were built up from consonant-vowel (CV) pairs. Accordingly, each short label had a CVCV and each long label had a CVCVCVCV structure. In the Abbreviated Label condition the first four letter of each long non-word was exactly the same as the short non-word within a label pair. In the Different Labels condition the label pairs were completely dissimilar (see Fig. 1). Half of the unfamiliar objects were frequent (or rare) in half of the participants, while the same objects were rare (or frequent) in the other half of the subjects, thus the visual features, for example the complexity of objects were counterbalanced between participants. The presentation side of short and long words was counterbalanced within participants.

### 2.4 Procedure

The participants were seated in front of the computer and the Button Box. First, they had to fill in and sign an informed consent form. Then, they were instructed to pay attention to the monitor to watch the series of pictures of the unfamiliar objects that were displayed in a consecutive manner. After the observational phase, the participants were informed that they will see each object once again accompanied with two words written in a foreign language. Their task was to guess, which label could be the name of the object on screen. Participants could indicate their answer by using the Button Box. When they selected a label for each of the twenty unfamiliar objects, the experimenter debriefed them about the aim and hypothesis of the experiment.

### 2.5 Data analysis

The dependent variable was whether a subject selected the long or short label to refer to a referent object. This data was analysed by SPSS 25.0 using binomial logit GLMM with Frequency (rare vs. frequent objects), Condition (Abbreviated vs. Different Labels), and Visual Features (see Stimuli for further explanation) as a fixed factor and Subject and Test Item as random factor. Since previous studies (e.g. Lewis & Frank, 2016) revealed an effect of object complexity on expected word length a geon-based Complexity score was assessed and it was also added to the GLMM model as a fixed factor. Complexity scores in the present study varied between two and six, while in the original study of Lewis and Frank (2016) it was between one and five. The performance of participants was also contrasted with chance-level using Wilcoxon signed-rank tests. Chance level was assumed to be 50% in line with previous studies using two alternative forced-choice tasks.

## 3 Results

**Fig. 2:**
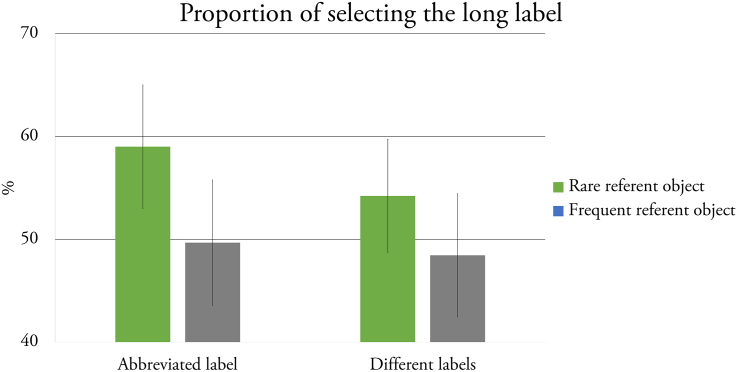
Average of selecting the longer label in the Abbreviated Label and Different Labels conditions. The error bars represent *SEM*.

The GLMM test revealed that Frequency (*F*(1, 3578) = 4.604, *p* = 0.032) and Complexity (*F*(1, 3578) = 8.048, *p* < 0.001) had a significant main effect on selecting the longer label. No other significant main effects or interactions were found. Wilcoxon signed-rank tests showed that the frequency of selecting the longer labels significantly differed from chance-level in the case of rare objects (*N* = 180, Z = −4.268, *p* < 0.001), both in the Abbreviated Label (*N* = 90, Z = −3.99, *p* < 0.001) and Different Labels condition (*N* = 90, Z = −2.499, *p* = 0.012), but not when participants' performance was tested with frequent objects (*N* = 90, Z = −1.422, *p* = 0.156) (Fig. 2).

## 4 Discussion

The results of the present study supported the main hypothesis as the participants selected the longer label for the rare object significantly more often in both the Abbreviated Label and the Different Labels conditions. This effect was significant when participants' choices were compared to the chance-level, however it is important to note that chance-level was arbitrary in the lack of a baseline measurement. The two distinct conditions showed a similar pattern of results, indicating that the main finding of the present experiment is robust. Moreover, the similar findings suggest that language users do not simply expect to abbreviate a word (Mahowald et al., 2013) when it refers to a frequent object, but may also have an implicit assumption that less frequent (and so less predictable) objects have longer names.

In line with the increasing number of studies showing that the linguistic context affects expectations about word length (Kanwal et al., 2017; Mahowald et al., 2013; Piantadosi et al., 2011) the present results suggest that language users take into account the referential-situational context as well. Since words and labels often refer to visual objects or events, the relevant characteristics of these referents are also important to monitor, especially when the label and the referent object is unfamiliar to the observer and the meaning of a signal should be inferred (see e.g. Tauzin & Gergely, 2018). This assumption fits to the pragmatic theories of communication (Grice, 1957; Sperber and Wilson, 1986, Sperber and Wilson, 2002), which hold that human languages rely heavily on inferential capacities to recognize the intended meaning of the communicator and the indicated referent by exploiting both linguistic and non-linguistic information sources to disambiguate communicative signals.

The present results further support this theoretical approach while extending previous empirical findings suggesting that language users can use the frequency of an object as a cue to identify whether a long or a short label refers to it. Assuming that communicative efficiency affects word length to be precise and concise at the same time, it can be raised that it is more efficient to refer to a frequent object with a shorter label than with a long one, as it may occur more often in speech as well. This mechanism can explain why “cellular phone” become “cell phone” or “West Highland White Terrier” often referred nowadays as “Westie”. It is also possible, however, that the effect found in the present study works the other way around. Rare objects in our environment are often specialized tools like an “electroencephalograph” having a name which refers to the properties or aim of that particular object. Accordingly, language users may expect that rare objects have to have longer names to be precise – in line with the results of the present study. Importantly, frequency and predictability were confounded in the present experiment unlike to previous studies (Mahowald et al., 2013; Piantadosi et al., 2011), therefore it is possible that differences in the predictability of objects – rather than differences in object frequency – led to different expectation in word length.

The implicit inferential cognitive mechanism that is hypothesized to track correlations between the frequency of a referent and label length might use statistical regularities represented in the mental lexicon. This can be preserved or even amplified by the intergenerational transmission of language during which the new words that are acquired by children will reflect the effect of communicative efficiency, therefore, shorter labels will be related to more frequent referents. It is an open and relevant question, however, whether there is a similar innate predisposition in infants, which can be investigated in future developmental studies. Nevertheless, since communicative efficiency serves to facilitate the informative function of language, words have to be dissimilar enough to avoid the confusion of referents (Dautriche, Mahowald, Gibson, & Piantadosi, 2016), consequently, it can be assumes that the effect of referent frequency on label length is limited.

A further interpretation of the present results is that information content of the referent predicted label length. Consequently, compared to previous works (e.g. Piantadosi et al., 2011) in the present task information content was not calculated from inter-word statistical dependencies, but assessed based on the frequency of an object. Accordingly, the results reported here can be interpreted in information theoretical terms (Shannon, 1948), namely that less frequent objects were paired with longer labels as both of these have higher levels of estimated information content. Hence, an object and the expected length of a label may be related. From this perspective, information content of an unfamiliar label would reflect that the appearance of a referent object is less likely, due to its low frequency.

Crucially, all the previously explained interpretations of the findings imply that frequency of a referent object has a direct effect on expected label length. Nevertheless, it is also plausible that the situational context modulates expectations about word form indirectly through the linguistic environment in which a given label is hypothesized to be embedded. Although the present paradigm – where unfamiliar labels were presented in the absence of the linguistic context – was not appropriate to measure such an effect, it can be assumed that certain situational and linguistic contexts are more likely to co-occur, therefore, label length might be influenced indirectly by the expected linguistic context. This account is in line with recent results, which provided evidence that the linguistic context can modulate word form (Mahowald et al., 2013; Piantadosi et al., 2011), but it may also extend previous approaches by raising the possibility that the situational context can change linguistic expectations and through that, the expectations about label length.

Another relevant aspect of the present experiment is that – although complexity scores could not be precisely measured here – the present study replicated a recent finding (Lewis & Frank, 2016), which shows that geon-based complexity of unfamiliar objects predicts expected word length. This may suggest that besides the frequency of a referent another relevant characteristic of it, namely its complexity can have an effect on label length. Nevertheless, this may only reflect an iconic relation between word form and meaning (Perniss et al., 2010) as complexity of unfamiliar objects – which is a highly salient feature of them – can only be expressed by adjusting word length if a new label has to be chosen or created. Consequently, it seems plausible to assume that the relation between object complexity and word length is independent from the frequency-based phenomenon revealed in the present study, however, depending on the available input both can be important when predicting language users behavior.

## 5 Conclusions

On the basis of this experiment it can be assumed that – at least in the case of novel referent objects and unfamiliar labels – language users expect a longer non-word to refer to a less frequent object. This finding suggests that the referential-situational context can have a direct (or indirect) effect when word meaning is inferred to understand and acquire novel communicative signals.
